# CODEX-aligned dietary fiber definitions help to bridge the ‘fiber gap’

**DOI:** 10.1186/1475-2891-13-34

**Published:** 2014-04-12

**Authors:** Julie Miller Jones

**Affiliations:** 1 St. Catherine University, Distinguished Scholar and Professor Emerita of Food & Nutrition, 4030 Valentine Ct. Arden, Hills, 55112, MN, USA

**Keywords:** Dietary fiber, Added fiber, CODEX fiber definitions, Fiber definitions and methods, Non-starch polysaccharide (NSP), Fiber benefits, Resistant oligomers and resistant starch, DP 3–9

## Abstract

A comprehensive dietary fiber (DF) definition was adopted by the CODEX Alimentarius Commission (CAC) (1) to reflect the current state of knowledge about DF, (2) to recognize that all substances that behave like fiber regardless of how they are produced can be named as DF if they show physiological benefits, and (3) to promote international harmonization for food labeling and food composition tables. This review gives the history and evolution of the state of DF knowledge as looked at by refinements in DF methods and definitions subsequent to the launch of the DF hypothesis. The refinements parallel both interventional and epidemiological research leading to better understanding of the role of DF in contributing to the numerous physiological benefits imparted by all the various digestion resistant carbohydrates. A comparison of the CODEX definition (including its footnote that authorizes the inclusion of polymers with DP 3–9) and approved CODEX Type 1 methods with other existing definitions and methods will point out differences and emphasize the importance of adoption of CODEX-aligned definitions by all jurisdictions. Such harmonization enables comparison of nutrition research, recommendations, food composition tables and nutrition labels the world over. A case will be made that fibers are analogous to vitamins, in that they vary in structure, function and amount needed, but each when present in the right amount contributes to optimal health. Since the intake of DF is significantly below recommended levels throughout the world, the recognition that ‘all fibers fit’ is an important strategy in bridging the ‘fiber gap’ by enfranchising and encouraging greater intake of foods with inherent and added DF. Fortifying foods with added DF makes it easier to increase intakes while maintaining calories at recommended levels.

## Introduction

Dietary fiber’s (DF) introduction as a concept in the latter half of the last century sparked the need to define DF and to develop a method that emulates the fate of these digestion-resistant materials and that characterizes its health-promoting roles. Defining DF has been both challenging and controversial for several reasons. First, DF can neither be defined as a single chemical entity nor group of related compounds. Second, different fiber types may have one or more physiological functions or health benefits. These may be similar or overlapping and, in some cases, may be unique for a particular fiber entity. Not all fibers perform all functions. So it is difficult to define it by health outcomes. Third, controversy swirls around whether DF has positive benefits only within the food matrix or whether it functions when isolated. Thus, numerous definitions have been proposed in order to address some of these various issues.

In 2009 CODEX published its DF definition, which resulted from nearly two decades of discussion among scientists and delegates from member nations. CODEX’s mission is to promote international harmonization, therefore it strives for a definition with worldwide acceptance [1,2]. Since the publishing of the CODEX definition, many countries have adopted aligned definitions [3-5]. The newly adopted definitions will increase the need for revisions to food composition tables and nutrition labels. Since the format and content of nutrition labels are under discussion and in some countries have been formally proposed, it is useful to consider labeled nutrients such as DF and to evaluate how differences amongst the various definitions affect labeled values.

The effect of various definitions also needs to be assessed in terms of the conduct and interpretation of research and addressing the ‘fiber gap’ [6-13].

DF intake in most countries around the world is far below recommended levels [11-13]. The gap between DF recommendations and intakes is so extreme that the U.S. Dietary Guidelines Advisory Committee (DGAC) listed DF as one of five ‘nutrients of concern’ [13].

All DF definitions, including the one not using the term DF, but rather uses ‘non-starch polysaccharides’ (NSP) [14], identify DF as carbohydrate (CHO) polymers and oligomers materials that escape digestion in the small intestine and pass into the large intestine, where they are slightly or nearly completely fermented. DFs *per se* and their fermentation products contribute to the many physiological benefits associated with their consumption. DF can directly influence the colon and microbiome, and its fermentation products can be absorbed from the large bowel to exert systemic influence in various parts of the body.

This review will give a brief history of the DF research, definitions and methods; compare and contrast the various DF definitions to the CODEX definition; provide justification for inclusion of DF materials that meet the definition including those with a degree of polymerization (DP) 3–9; underline the need for international harmonization in DF research, food composition tables and food labeling; outline the alarming gap between recommended and actual intakes; suggest that a balance of fiber types is important- making DF analogous to vitamins; and give rationale for including all three CODEX categories of DF - intrinsic, added and synthesized or modified for addressing the “fiber gap” and improving human health.

## Review

### Evolution of the DF definition

In 130 A.D. the physician Galen unknowingly referred to DF when he wrote about foods that “excite the bowels to evacuate and those that prevent them” [15]. He noted that white bread is “the stickiest and slowest to pass and that brown bread is good for the bowels”. From this moment on, the undigested material that ‘excites the bowels’ has been associated with gut health.

In the first part of the last century, studies of non-digestible materials in animal studies assessed their impact on utilization of macronutrients [16], but the term ‘dietary fiber’ was not used until the 1940s [17]. In human nutrition, the term ‘DF’ was first used in a study of pregnant women where it was observed that those eating diets high in DF had a lower incidence of toxemia [18].

Until the 1970s, nutrition textbooks covered DF in a single paragraph that noted it was a CHO that resisted digestion and improved laxation. Measurement at that time was part of the proximate composition analysis and was reported as crude fiber (CF). This changed with observations in the late 1960s and early 1970s by British physicians working in rural Africa. They observed that diseases common in the West were rare, and proposed that the observed differences were due to the unrefined nature of African diets. Thus, the DF hypothesis, suggesting that undigested CHO could reduce chronic disease, was launched [19-21].

#### The search for a DF definition, methods and role

The DF hypothesis spurred a quest for methods and a definition that would adequately characterize this material in food and emulate conditions in the digestive tract. It also launched many research projects looking at its health benefits. The only existing DF method, the CF method, was found wanting because its strong solvents and harsh conditions failed to approximate DF’s fate in the digestive tract [22-25].

Interest in DF’s physiological role stimulated both a re-examination of older intervention studies and new research, especially epidemiological research, which tried to characterize the relationship of DF to various health conditions. Existing studies, most with isolated fibers, indicated DF’s beneficial effects on laxation, gut health, nutrient availability, enzyme activity, and cholesterol or atherosclerosis reduction [26-28].

#### Current DF definitions and methods

Table 1 gives some DF definitions from around the world including those from CODEX, the U.S. Institute of Medicine (IOM), Health Canada, European Food Safety Authority (EFSA), Food Standards Australia and New Zealand (FSANZ), and American Association of Cereal Chemists International (AACCI) and fiber as described as NSP [1,3-6,14,29].

**Table 1:** Current operative definitions for dietary fiber from around the world

**Organization**	**Definition**
*CODEX Alimentarius Commission* 2009 (Sets International guidance standards for food and food imports).	•Dietary fiber means carbohydrate (CHO) polymers with ten or more monomeric units^1^, which are not hydrolyzed by the endogenous enzymes in the small intestine (SI) of humans and belong to the following categories:
	•Edible CHO polymers naturally occurring in the food as consumed
	•CHO polymers, obtained from food raw material by physical, enzymatic, or chemical means^2^
	•Synthetic CHO polymers^2^
	○^1^The footnote allows international authorities to decide whether those compounds with DP of 3–9 would be allowed.
	○^2^ For the isolated or synthetic fibers in category ‘2’ or ‘3’ , they must show a proven physiological benefit to health as demonstrated by generally accepted scientific evidence to competent authorities
	***Includes resistant oligosaccharides, resistant starch and resistant maltodextrins when footnote 2 is included.***
*Health Canada (HC)*2010 (A department within the Canadian government responsible for national public health).	•Dietary Fiber Consists of naturally occurring edible carbohydrates (DP > 2) of plant origin that are not digested and absorbed by the small intestine and includes accepted novel dietary fibers.
	•Novel Dietary fiber is an ingredient manufactured to be a source of dietary fiber. It consists of carbohydrates (DP > 2) extracted from natural sources or synthetically produced that are not digested by the small intestine. It has demonstrated beneficial physiological effects in humans and it belongs to the following categories:
	•Has not traditionally been used for human consumption to any significant extent, or
	•Has been processed so as to modify the properties of the fiber, or has been highly concentrated from a plant source
	***Includes resistant oligosaccharides, resistant starch and resistant maltodextrins.***
European Food Safety Authority (EFSA) 2009 (The Panel on Dietetic Products, Nutrition and Allergies develops scientific opinions on reference values for the European Union).	•Non-digestible carbohydrates plus lignin, including all carbohydrate components occurring in foods that are non-digestible in the human small intestine and pass into the large intestine
	***Includes non-starch polysaccharides, resistant starch, resistant oligosaccharides.***
Food Standards Australia and New Zealand *(FSANZ)* 2001 (Responsible for development and administration of the food standards code listing requirements for additives, safety, labeling, and genetically-modified foods).	•Dietary fiber means that fraction of the edible part of plants or their extracts, or synthetic analogues that:
	•Are resistant to digestion and absorption in the, usually with complete or partial fermentation in the large intestine; and
	•Promote one or more of the following beneficial physiological effects:
	○laxation
	○reduction in blood cholesterol
	○modulation of blood glucose
	***Includes resistant polysaccharides, oligosaccharides (DP >2) and lignins and resistant starches.***
American Association of Cereal Chemists *(AACC)* 2001 (Gathers scientific and technical data for global use by grain-industry professionals; currently know as AACCI).	•The edible parts of plants or analogous CHOs’ that are resistant to digestion and absorption in the human small intestine, with complete or partial fermentation in the large intestine
	•Dietary fiber includes polysaccharides, oligosaccharides, lignin, and associated plant substances.
	•Dietary fibers promote beneficial physiological effects including laxation, and or blood cholesterol attenuation, and/or blood glucose attenuation.
	***Includes resistant oligosaccharides, resistant starch and resistant maltodextrins.***
Institute of Medicine *(IOM)* 2001 (U.S. and Canadian advisory organization of the National Academy of Sciences; provides science- based research and evidence-based analysis to improve national health).	•*Dietary Fiber* consists of non-digestible CHOs’ and lignin that are intrinsic and intact in plants.
	•*Functional Fiber* consists of isolated, non-digestible CHOs’ w/ beneficial physiological effects in humans.
	•*Total Fiber* is the sum of Dietary Fiber and Functional Fiber.
	***Includes resistant oligosaccharides, resistant starch and resistant maltodextrins.***
NSP Non-Starch Polysaccharides	•The skeletal remains of plant cells that are resistant to digestion by enzymes of man measured as non α-glucan polymers measured by the Englyst (Type 2 Method).
	•It includes NSP, which is comprised of cellulose, hemicelluloses, pectin, arabinoxylans, beta-glucan, glucomannans, plant gums and mucilages and hydrocolloids, all of which are principally found in the plant cell wall.
	***Does not include oligosaccharides, resistant starch and resistant maltodextrins.***

The 2009 Codex definition resulted from a nearly two-decade process undertaken by CODEX Alimentarius Commission (CAC), whose mission it is to provide global guidance and harmonization about matters regarding food and international trade [1,2]. (Table 2 gives a brief overview of the CAC.) The definition and its two footnotes read as follows:

*Dietary fibre means carbohydrate polymers*^*1*^*with 10 or more monomeric units*^*2*^*, which are not hydrolysed by the endogenous enzymes in the small intestine of humans and belong to the following categories:*

1. *Edible carbohydrate polymers naturally occurring in the food as consumed.*

2. *Carbohydrate polymers, which have been obtained from food raw material by physical, enzymatic or chemical means and which have been shown to have a physiological effect of benefit to health as demonstrated by generally accepted scientific evidence to competent authorities,*

3. *Synthetic carbohydrate polymers, which have been shown to have a physiological effect of benefit to health as demonstrated by generally accepted scientific evidence to competent authorities.*

Footnote 1 states, “when derived from a plant origin, dietary fibre may include fractions of lignin and/or other compounds associated with polysaccharides in the plant cell walls. These compounds also may be measured by certain analytical method(s) for dietary fibre.

Footnote 2 states that, “Decision on whether to include carbohydrates of 3 to 9 monomeric units should be left up to national authorities.”

**Table 2:** Codex alimentarius commission in brief

•	Established in 1963 as part of **the World Health Organization Food and Agriculture Organization (WHO/FAO)**
	○ Covers 180 countries
	○ Represents 99% of the world’s population
•	Formed to set safety, quality and fairness for international food trade
	○ Sets international food standards
	○ Gives guidelines and codes of practice for labeling and food and agricultural processes
•	Aims to **achieve international harmonization** in quality and safety requirements

The two footnotes require comment. Footnote 1 aligns with most definitions and recognizes ‘associated substances’ and lignin when part of, but not isolated from, the DF complex.

#### The CODEX definition and footnote 2

Footnote 2 allows national authorities the option of including digestion-resistant oligomers with DP 3–9, thus enabling different operative definitions of DF. This is counter to CODEX’s mission, which is to facilitate international harmonization for food labeling, food composition tables, and interpretation of research. For example, results of studies including DP 3–9 could differ from those excluding them. DF values for foods such as wheat and onions that contain digestion-resistant oligomers with DP 3–9 or resistant starch (RS) could differ on food labels and in food composition tables. Thus, epidemiological research using food composition tables might also differ if some countries include these materials and others do not.

The exclusion of resistant CHOs with DP < 10 is an artifact of early DF methods and is not helpful for two reasons. First, an initial alcohol wash elutes short-chained fibrous materials. The loss of this short-chain, non-digestible material is not based on differences in physiological impact or data showing different health outcomes, but merely differences in solubility. Second, the alcohol separation has been shown to lack a precise cutoff at DP = 10, and may elute materials with DP > 10 [25]. Thus, the arbitrary cut-off at DP 10 does NOT have a sound basis either analytically or physiologically.

Resistant short-chain oligomers (DP 3–9) should be DF because they fit the definition in the following ways: 1) they are neither digested nor absorbed by the enzymes in the small intestine, 2) they are fermentable in the large intestine, 3) they aid laxation in the large bowel, and 4) they may increase mineral absorption [30-32]. If these materials are not included as DF, they would be cast into a ‘no-man’s land’ of being neither a digestible CHO nor DF [33]. DF experts from around the world attending the 2010 International Vahouny Dietary Fiber conference voted overwhelmingly for the inclusion of all indigestible CHO oligomers and polymers with DPs of 3 or higher [34].

In the spirit of international harmonization, many experts recommend the acceptance of the entire CODEX definition including footnote 2, which accepts all digestion-resistant polymers with a DP > 3 [35]. To that end many jurisdictions have adopted the CODEX-aligned definitions in its entirety. Table 3 contains a list of regions that have definitions aligned with the entire CODEX definition.

**Table 3:** Countries adopting the CODEX with DP >3 dietary fiber definition

**Authorities/Countries accepting the definitions with DP3**	**Countries not accepting the definition with DP3**	**Countries awaiting a decision**
EFSA/European Union	South Africa	US FDA
Food Standards Australia and New Zealand (FSANZ)		
Brazil		
Health Canada		
Chile – for labeling	Chile - not for health claims	
China		
Indonesia		
Korea		
Malaysia		
Mexico		
Thailand		

### ‘All fibers fit’

All DF definitions recognize non-digested fibrous materials inherent in food as part of the fiber complex. Most definitions also enfranchise these non-digested CHO materials, when they are extracted from edible material, synthesized, or modified, IF they have at least one proven health benefit.

Determination of health benefits requires consideration of both epidemiological and intervention studies because each study type has limitations. Intervention studies are conducted with a specific DF (or DF blend) are usually limited in duration, have few subjects and require a measurable disease biomarker. Subjects in the small pool may not be at risk for the endpoint being measured. Further, the study’s short duration or design may fail to document the impact of DF on disease because of the inability to assess the effects of habitual intakes or to show synergies with other dietary components [36,37]. Epidemiological studies, on the other hand, have the advantage of huge numbers of subjects with a wide range of disease susceptibility. However, they may be subject to confounding as those with high DF intakes frequently have a number of healthy lifestyle and dietary patterns [38]. Further, dietary intake instruments often fail to accurately characterize habitual diets or food preparation effects. The analysis of epidemiological data relies on food composition databases, which have not been updated to include short-chained oligomers and RS or to have accurate data on DF added to food [39,40].

### The various definitions: similarities and differences

While the various DF definitions show many similarities, some important differences exist (Table 1). All definitions enfranchise CHO polymers that resist digestion and absorption in the human small intestine. However, the NSP definition only recognizes plant cell wall fibers, and does not include synthetic, resistant CHO polymers or polymers extracted from raw food material by physical, enzymatic or chemical means.

The IOM definition [6] bears similarity to CODEX-aligned definitions in that it accepts that all resistant carbohydrate materials whether intrinsic or extracted or synthesized. However, it reserves the term ‘dietary fiber’ only for materials that are intrinsic and intact within food. Extracted, modified or synthesized fibers are defined as ‘functional fiber’. The sum functional and intrinsic DF is ‘total fiber’, rather than DF as in all other definitions except the NSP definition [6,14].

While difference between the IOM and the CODEX definition is small, it may create analytical challenges and cause confusion for several reasons. First, the term ’DF’ is used in labeling and nutrient databases and nutrition research, where fibers from foods and fibers added to the diet are usually considered collectively and not separately. Second, the two IOM categories cannot be differentiated analytically when the food inherently contains the fiber that is being added (e.g. an oatmeal muffin with added β-glucan) [24,25]. Third, not referring to both types as DF is inconsistent with the reporting of other nutrients in food composition tables, food labels and nutrient intake surveys. For other nutrients, reported values are the sum of that nutrient irrespective of whether it was added or inherent. Thus, the small nuance of difference between the IOM definition and other CODEX-aligned definitions could cause unintended points of difference.

Most definitions require that at least one physiological benefit be shown for fibers added back to food, e.g. those fibers in CODEX categories 2 or 3. Some definitions list specific physiological effects, as did all iterations of the CODEX definition except the final one [1,3-5,24]. These were: 1) improved intestinal transit time and increased stool bulk; 2) fermentation by colonic microflora; 3) reduction in blood total and/or LDL cholesterol levels; and 4) reduction in post-prandial blood glucose and/or insulin levels. Other definitions may include other physiological effects [3-5,24].

Most DF definitions accept Codex Footnote 1 and agree with the inclusion of lignin and associated substances as DF, but only when part of the native fiber complex. Some definitions allow only CHO from plants, while others recognize DF from animals (e.g. chitin). (Whether included or not, such fibers are captured by current analytical methods.)

Like the CODEX DF definition, many definitions include RS and resistant oligomers [1,3-5,24]. The AOAC 2009.01 (AACCI 32–45), a CODEX Type 1 method, best matches the CODEX definition and captures these digestion-resistant entities [2,39-43]. However, many food databases report values obtained using older methods. The NSP definition and method (not a Type 1 method) fail to capture resistant oligomers or RS [14,44]. (Note: The CAC strives to have a single Type 1 method with the imprimatur of AOAC International and the AACCI because of the rigorous collaborative testing in labs around the world prove them to be both rugged and reproducible. The NSP method is defined by CODEX as an empirical method, rather than a rationale method, and does not get full CODEX imprimatur as the method of choice).

### DF recommendations

DF intake recommendations for adults range from 18–38 g/d [3-6,45-48] (Table 4). WHO/FAO and EFSA recommend 25 g/d with their recommendations based on amounts needed for healthy laxation [5,46]. Some countries such as Singapore, the U.S. and Canada tie DF requirements to caloric intake, so recommended amounts for men, women and elderly vary. Thus, recommended levels are 25 g/d for women and 38 g/d for men in the U.S. and Canada. These are among the highest recommended levels not only because they are tied to calories, but also because they are based on the median intake associated with the lowest risk of coronary disease in prospective cohort studies [45]. The 18 g/d recommended by the UK Food Standards Agency is lower than most other recommendations and reflects the use of NSP definition and methods rather than the CODEX definition and method [47]. This is an example where confusion is caused by different jurisdictions using non- CODEX aligned definitions and methods [2].

**Table 4:** Adult fiber recommendations and average intakes in selected countries

**Country/Region**		**Recommended fiber intake (g/day)**	**Median intake (g/day)**	**Body issuing the requirement**
US and Canada	Males	38	16.5-19.4	North America – Jointly use the IOM report from the National Academy of Sciences
	Females	25	12-15	
France	Males	30	21	*Agence française de sécurité sanitaire des aliments* (French food safety agency)
	Females	25	17	
Germany	Males	30	24	German Nutrition Society
	Females	30	21	
Japan	Males	30	17	Japanese Ministry of Health
	Females	25	17	
UK	Males	18*	15.2	UK Department of Health
	Females	18*	12.6	
FAO/WHO		>25		WHO/FAO
		>20		

*Lower requirements due to use of the NSP method.

#### DF intakes

There is a concerning gap between recommendations and intake worldwide [11-13,49]. For example in North America, the average daily consumption of adults is 15 g/d, which means that those in the lowest quintiles of intake ingest below half the recommended levels [50-52]. Evaluation of NHANES 2003–2006 data show that under 5% of the US population ingests the recommended intake [11,12].

A few initiatives have been successful in reducing the DF gap, such as programs from the Grains & Legumes Nutrition Council (formerly GoGrains) in Australia and New Zealand [53]. However, in most countries intake has not increased, despite a consistent body of evidence associating DF with reduced health risks [54-64] and messages from health promotion organizations to increase DF intake for both prevention and management of disease [13,65-67]. Food intake surveys in the U.S. show that intake has remained roughly the same for over nearly two decades [11,12,50]. The DF gap even occurs in children in many countries and manifests itself as chronic constipation [68,69]. Inadequate intake of DF in childhood is thought to be associated with health risks including obesity in later life [70].

#### “All fibers fit” to address the fiber gap

Adoption of CODEX-aligned definitions provides a strategy to help address the fiber gap by acknowledging the role of both intrinsic and added fibers. The ‘all fibers fit’ mantra means that healthy diets include the various fiber types [31]. Constructing a diet with a balance of DF types is analogous to constructing a diet with the correct amount and balance of the vitamins needed for optimal health. The similarities are outlined as follows. First, vitamin requirements are fulfilled only when all vitamins are present both in quantity and type. In like manner for DF, the optimal diet would have both right quantity of total DF and balance of fiber types. Second, vitamins inherently in food and those added through fortification and enrichment can work together to fulfill the requirements [71,72]. The same can apply to DF in that added fibers can interact with intrinsic fibers to meet the requirement act synergistically actually to reap health benefits.

Variety is one of the important concepts of healthy diets. Just as a balanced diet incorporates adequate amounts of each vitamin and avoids excesses of any one kind, an optimal diet should incorporate the right amount and balance of fiber different types needed to perform DFs’ many functions. Thus a varied diet that uses fiber-fortified foods together with fiber-rich foods has multiple benefits. It ensures inclusion of associated substances that are trapped in the DF matrix of natural foods, allows health benefits from the synergy of DF types and functions, and the fiber gap while staying within a day’s calorie allotment.

Efforts to increase the intake of fiber-rich foods - whole grain breads and cereals or pseudocereals (wheat, maize, oats, rye, barley, triticale, millet, sorghum, buckwheat, etc.), legumes and soy, fruits, vegetables, nuts, and seeds - must continue. While diets constructed using USDA MyPlate and DGAC recommendations can attain DF levels as high as 50 g/d with the selection of high-fiber options within and among the food groups (Table 5), analysis of food consumption data show that most consumers chose low-fiber foods frequently. Since a serving of many common foods provides 1–3 g of DF, individuals eating according to the MyPlate guidelines *could* ingest on average 20–24 g/d, but might ingest as little as 12–15 g/d. However, only 3-8% of the US population eats according to USDA MyPlate, and the fiber-containing food groups are most frequently omitted [13,50-52,73-81].

**Table 5:** High, low and average DF for consumers meeting myplate servings

		**Average and range of fiber content**			**Diets daily totals of diets constituted with**		
**Food group**	***Recommended servings per MyPlate section (2000 cal diet)***	***Average fiber per food in the MyPlate group***	***Low fiber perfood in the MyPlate group***	***High fiber per food in the MyPlate group***	***Total fiber per MyPlate category with foods choices with low fiber content***	***Total fiber per MyPlate category with foods choices with average fiber content***	***Total fiber per MyPlate category with foods choices with average fiber content + 1 fiber-fortified food***
		Grams fiber/d					
Vegetables	5 ½ cup	2.5	1	6	5	12.5	18
Fruit	3 ½ cup	2.5	1	5	3	7.5	12
Protein	5.5 oz	<1	0	8	0	0	8
Milk	3 cup	0	0	1	0	0	1
Whole grain	3-1 oz equiv	2.5	1	11	3	8	17
Refined grain	3 1-oz equiv	1.5	<1	3	3	3	3
Totals					14	31	59

NHANES data from 2001–2004 document that under 5% of consumers in most age and gender categories meet the adequate intake (AI) for fiber [12]. Less than 10% of the population meets the whole grain recommendation [80,81]. Fruit and vegetable intakes are about half that recommended, and again the higher-fiber choices within each groups are selected less frequently [81]. Per capita consumption of legumes is extremely low at 25 g/d (0.1 cup/d or 20 calories/day) [81].

Addressing the ‘fiber gap’ not only requires a continuing push towards greater intake of fiber-rich foods and the use of more foods with added fibers, but also needs to include consumer education about fiber sources. Surveys show that consumers are looking for more DF, but also reveal that they have difficulty choosing foods or groups of foods that are DF sources, identifying isolated fibers on the ingredient statement, or estimating the DF content of foods and diets [82-84].

Statements by some health professionals and in corporate advertising campaigns suggest that added fibers are ‘fake’, and that only foods with natural fiber can fulfill DF requirements. While it is true that added fibers may not carry all the ‘co-passengers’ found in the food fiber matrix, isolated fibers do show documented health benefits [85]. In fact these benefits are so well documented that many countries allow health claims for isolated fibers such as wheat bran and β-glucan and its derivatives. In some cases isolated fibers and their derivatives are more concentrated both in terms of DF and trapped co-passengers enabling intake of a smaller amount of food for an equivalent physiological dose than would required from the whole food. For example, the addition of oat bran and β-glucan to a serving of oatmeal yields a cholesterol-lowering dose of DF with half the calories of an equivalent dose of oatmeal without added DF [86].

The synergy of fiber inherent in foods and that added to foods has been documented and needs to be emphasized [87-89]. For example, a meta-analysis of intervention studies suggests that type 2 diabetes risk reduction is reached either with isolated fiber or with DF from whole foods [90]. Improved blood lipids and weight management was shown to occur with isolated fiber added to a diet with a baseline of 20 g of DF. Discounting of the contribution of added fibers because of their lack of epidemiological evidence fails to consider studies such as this cited meta-analysis [90].

Teasing out the effects of isolated DFs from epidemiological studies is methodologically challenging because food databases used in the analysis of food frequency only report total DF and do not attribute DF source. Thus health impacts of added fibers may be not be captured. Further, food composition data may not reflect added fibers especially those that are short-chain oligomers and resistant starch, so their presence may be underestimated [91]. Thus, epidemiological studies attribute all health benefits to DF that are inherent in foods and fail to recognize the contribution of added fiber or its synergy in impacting health and disease. However, data from intervention studies clearly show that increases in total DF intake through fibers added to a baseline of diet rich in whole grains, fruits and vegetables, legumes and other fiber-rich foods synergistically improves health outcomes over those observed with lower fiber intakes [87-89].

## Conclusions

Worldwide there is a ‘fiber gap’ because intake is far below the recommendations. The gap is so extreme that the 2010 U.S. DGAC followed previous committees in naming DF as one of five ‘nutrients of concern’. Less than one in ten Americans meets the fiber requirement.

The new CODEX definition enfranchising added fibers along with fibers traditionally found in food underscores the need for all types of fibers – those which naturally occur in vegetables, fruits, whole grains, nuts, seeds and legumes and those that are isolated or synthesized and added to foods - as part of a strategy to address the inadequate intake of DF. The new definition reflects advances in the knowledge of dietary fiber types and roles and shows that all CHOs with DP > 3 that resist digestion and that have a beneficial physiological effect are included and include them even though certain earlier methods did not necessarily capture these materials. Acceptance as a DF is based on proof of a physiological benefit.

With CODEX-aligned definitions across jurisdictions, the development of great-tasting, high-fiber foods by the food industry is facilitated. Consumer education programs can heighten awareness of the ‘fiber gap’ and suggest workable dietary strategies to address the problem. This can include programs that help consumers identify isolated DFs, choose foods naturally rich in fiber, and model diets which achieve fiber recommendations with a combination of foods naturally high in fiber and those with added fiber. Such models can show consumers how to increase fiber intake while helping consumers to stay within their calorie budget and encourage them to act on the principle laid out in the CODEX definition that ‘all fibers fit’.

In summary, adoption of CODEX-aligned definitions and approved methods, which include resistant oligomers DP 3–9 (included in Footnote 2) and resistant starches, is needed in all jurisdictions because such an action recognizes the current state of fiber research and methodology, facilitates the conduct of nutrition research that is comparable, harmonizes nutrition labeling and food composition tables, and provides a platform to educate consumers about the benefits of dietary fiber and risks of not choosing it. A single voice with consistent messaging about the benefits of DF can help consumers build diets that will meet intake goals to address the ‘fiber gap’ and reap the benefits of diets that have increased DF.

## Abbreviations

AACCI: American Association of Cereal Chemists International; AOAC: Association of Analytical Chemists; CAC CODEX: Alimentarius Commission; CF: Crude fiber; CHO: Carbohydrate; DF: Dietary fiber; DGAC: Dietary Guidelines Advisory Committee; DP: Degree of polymerization; EFSA: European Food Safety Authority; FAO: Food and Agriculture Organization; FSANZ: Food Standards Australia and New Zealand; FDA: US Food and Drug Administration; ILSI: International Life Sciences Institute; IOM: Institute of Medicine; NSP: Non-starch polysaccharides; RS: Resistant starch; WHO: World Health Organization.

## Competing interests

Funds to help with the preparation of this manuscript were given by the Calorie Control Council. In the past 5 years, I have given speeches and written articles where travel grants and honoraria are received from the food industry some of which produce or use whole grains and fibers such as ADM, Beneo, Campbell Soup Company, Ingredion, Kraft Foods, Proctor & Gamble, Tate & Lyle and Uncle Ben’s. I am a scientific advisor to the carbohydrate committee of the International Life Sciences Institute – North America, the WK Kellogg Corporation, the Quaker Oats Company, the Joint Institute of Food Safety and Applied Nutrition of the University of Maryland and the US Food and Drug Administration.

## Author’s information

I have worked on the AACCI committee looking at the dietary fiber definition. I was part of an ILSI team presenting at the Vahouny Dietary Fiber conference. I have done much work in the area of carbohydrate nutrition, resistant starch, sugars, whole grains and dietary fiber. I presented at a symposium prior to a CODEX meeting on definitions and methods for dietary fiber.
